# Hepatocellular Carcinoma in β-Thalassemia Patients: Review of the Literature with Molecular Insight into Liver Carcinogenesis

**DOI:** 10.3390/ijms19124070

**Published:** 2018-12-17

**Authors:** Antoine Finianos, Charbel F. Matar, Ali Taher

**Affiliations:** Division of Hematology/Oncology, Department of Internal Medicine, American University of Beirut Medical Center, Beirut 11-0236, Lebanon; af72@aub.edu.lb (A.F.); cm46@aub.edu.lb (C.F.M.)

**Keywords:** hepatitis C virus, hepatitis B virus, hepatocellular carcinoma, iron overload, thalassemia, screening

## Abstract

With the continuing progress in managing patients with thalassemia, especially in the setting of iron overload and iron chelation, the life span of these patients is increasing, while concomitantly increasing incidences of many diseases that were less likely to show when survival was rather limited. Hepatocellular carcinoma (HCC) is a major life-threatening cancer that is becoming more frequently identified in this population of patients. The two established risk factors for the development of HCC in thalassemia include iron overload and viral hepatitis with or without cirrhosis. Increased iron burden is becoming a major HCC risk factor in this patient population, especially in those in the older age group. As such, screening thalassemia patients using liver iron concentration (LIC) measurement by means of magnetic resonance imaging (MRI) and liver ultrasound is strongly recommended for the early detection of iron overload and for implementation of early iron chelation in an attempt to prevent organ-damaging iron overload and possibly HCC. There remain lacking data on HCC treatment outcomes in patients who have thalassemia. However, a personalized approach tailored to each patient’s comorbidities is essential to treatment success. Multicenter studies investigating the long-term outcomes of currently available therapeutic options in the thalassemia realm, in addition to novel HCC therapeutic targets, are needed to further improve the prognosis of these patients.

## 1. Introduction

Thalassemia is a genetic disorder characterized by quantitative disorder of hemoglobin resulting in an imbalance between the alpha and β chains. The end result of defects in the alpha and β globin genes is an imbalance between both chain types resulting in ineffective erythropoiesis and anemia. The degree of mutations in the β-globin genes dictates the phenotypic expression of β-thalassemia in patients where, while they were divided into thalassemia intermedia (TI) and major (TM), the degree of ineffective erythropoiesis divides these patients into transfusion-dependent (TDT) and nontransfusion-dependent thalassemias (NTDT) [1]. Ineffective erythropoiesis and subsequent hemolytic anemia results in decreased hepcidin and consequently results in increased iron absorption from the gut. Along with the need for frequent transfusions to ameliorate the resulting anemia, this results in an iron-overload state that can result in various types of organ damage, like heart failure, cirrhosis, and various endocrinopathies [2].

Hepatocellular carcinoma (HCC) was not as common in the past as it is now in thalassemia patients, as thalassemia patients used to die younger from anemia and cardiac failure related mainly to iron overload [3]. With the advent of effective iron chelation, prolongation of survival in thalassemia patients, especially due to prevention of cardiac disease, was possible, and thus these patients live long enough to develop long-term complications like HCC [4,5]. In this review, we discuss HCC in thalassemia patients in terms of epidemiology, disease characteristics, risk factors, prevention, and management of this comorbidity. We also shed light on various proposed mechanisms for liver carcinogenesis in thalassemia patients with any of the known risk factors (iron overload, hepatitis viral infection, cirrhosis). 

## 2. HCC in Β-Thalassemia—Incidence and Characteristics

The estimated incidence of new cases of liver and intrahepatic biliary tract cancers in the United States in 2018 is 42,220 cases. The estimated mortality is 30,200 cases, fifth after the lung, colon, pancreas, and breast cancers [6]. The average age at onset of HBV-related hepatocellular carcinoma (HCC) is around 50 years old, while that of HCV-related HCC is around 61–64 years old [7]. The median age of HCC onset in the U.S. general population is approximately 70 years, with a predominant male to female ratio of 2.4 to 1 [8,9]. Patients with genetic hemochromatosis were found to be 23 times more likely to have liver cancer than those without with an annual incidence rate of HCC-related cirrhosis due to genetic hemochromatosis of around 3%–4% [10]. Especially for those born before the 1990s, β-thalassemic patients that were periodically transfused were often infected with either hepatitis B (HBV), hepatitis C (HCV), or both [11]. Despite the introduction of deferoxamine in the late 1970s, patients with TDT continued to die in their second or third decades from cardiac complications, with very few surviving long enough to develop long-term complications like HCC. In fact, until 2002, only two cases of HCC in TM patients were reported [12,13]. Survival in thalassemic patients has been increasing [14]. This is majorly due to the better screening of blood products for hepatitis viruses, improved iron chelation, and possibly improved hepatitis B vaccination and hepatitis-virus eradication, the result of which is the noted increase in cases of HCC in thalassemic patients in the years from 1993 to 2012 [15]. Since 1986, around 93 cases of HCC in TM or TI have been reported in the literature [12,13,15,16,17,18,19,20,21,22,23,24]. In their initial report in 2004, Borgna-Pignatti reported 22 cases of HCC from 52 Italian centers with a mean age of 45 years (33–64 years) at diagnosis [17]. In their updated data in 2014, between 2002 and December 2012, 60 new cases of HCC were identified out of the 5855 thalassemia patients followed by the 55 participating centers. Cumulative incidence of HCC was reported to be 1.02%: 32 out of 4248 TM with an incidence of 0.75%, and 28 out of 1607 TI with an incidence of 1.74% [15]. Mancuso et al. conducted a prospective study to detect HCC incidence by screening B-thalassemia patients with liver ultrasound; 38 with TM and 70 patients with TI were evaluated [22]. In the study, two of the 72 patients with risk factors (iron overload and HCV) developed HCC and both have TI, while none of the 33 patients without any of the risk factors developed HCC. The incidence of HCC in this study was 2%. TI patients, and more specifically NTDT patients, seem to be at a higher risk of developing HCC, likely because of the improved survival, delayed use of iron chelation, and prolonged duration of iron overload and possible hepatitis-virus infections when compared to TM or TDT patients [5]. These risk factors, as discussed below, seem to play a major role, either alone or in combination, in the development of HCC in thalassemia patients.

## 3. HCC in Thalassemia Risk Factors: Hepatitis B/Cirrhosis

**Figure 1:**
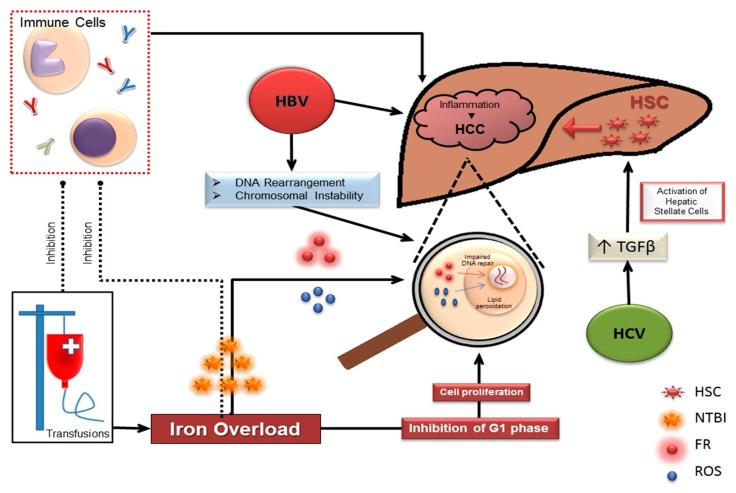
Different mechanism related to HCC development in thalassemia patients. FR: Free Radicals; HBV: Hepatitis B Virus; HCV: Hepatitis C Virus; HCC: Hepatocellular carcinoma; HSC: Hepatic Stellate Cells; NTBI: Non-Transferrin Bound Iron; ROS: Reactive Oxygen Species; TGFβ: Transforming Growth Factor β.

More than 50% of worldwide HCC cases are due to chronic HBV infection, with the highest HCC incidence in Asia Pacific and sub-Saharan Africa [25,26]. More than 70% of HBV-infected patients with HCC have underlying cirrhosis, with an annual incidence of HCC around 2.5% [27]. The exact oncogenic mechanism of HBV in general and in thalassemic patients remains to be determined, but likely related directly to HBV DNA [28]. Intracellular DNA rearrangement, enhancement mutagenesis, inflammation, and insertion of HBV oncogenic genes in the host chromosome leading to chromosomal instability are among some of the described oncogenic mechanisms [29]. Hepatic cirrhosis caused by persistent inflammation is a precursor for the development of HCC [30,31]. Continuous hepatic inflammation caused by the presence of the virus results in increased oxidative stress, repeated cycles of apoptosis, and compensatory hepatic regeneration. This may result in increasing the possibility of defective regulatory systems of apoptosis and subsequently immune surveillance, especially of premalignant hepatocytes [29]. Liver cirrhosis can be considered a chronic inflammatory state in the liver that can drive more fibrosis and create a proper milieu for HCC development [32]. Cirrhosis, which is the ultimate complication of HBV infection, was shown to be associated with epigenetic modifications resulting in the silencing of tumor-suppressor genes that may accelerate hepatic tumorogenesis [33,34]. Worldwide, 0.3% to 5.7% of thalassemia patients are HBsAg-positive [15]. In their series of thalassemic patients with HCC, Borgna-Pignatti et al. [17] reported two TM patients with HBs Ag+, one with associated HCV RNA+ and the other with HVC AB+, but negative HCV RNA status. Moreover, both had elevated serum ferritin at diagnosis (2950 and 1524 µg/L) and had HCC at a relatively young age (33 and 35). In their updated data in 2014, Borgna-Pignatti reports three out of 62 HCC patients with HBsAg+ disease [15]. The paucity of reported cases of HCC in thalassemic patients with HBV is likely related to the fact that the majority of reports come from developed countries or countries where HBV incidence is low due to the effective control of risk factors, but also effective HBV-related vaccinations and treatment. (Figure 1)

## 4. HCC in Thalassemia Risk Factors: Hepatitis C

In contrast to HBV, HCV is more common in developed countries [35]. Similar to what is described for HBV above, HCV-mediated carcinogenesis is either directly related to the virus or to complications of HCV hepatic infection like fibrosis and cirrhosis. HCV genes seem to induce the production of transforming growth factor β and consequently activating hepatic stellate cells that are responsible for hepatic fibrosis [36]. The prevalence of anti-HCV antibodies in thalassemic patients ranges from 4.4% to 85.4% [37]. Of the HCC in thalassemia reported in the literature, 88% are infected with HCV (either as HCV Ab or HCV RNA). In the initial Italian registry, out of 22 patients with HCC and thalassemia, 86% were HCV+, while in the updated data, out of 62 thalassemic patients with HCC, 54 were HCV+, accounting for around 87% of cases [15,17]. Even in the absence of significant cirrhosis, HCC may still occur in thalassemic patients. Mancuso et al. [22] reported HCC occurring in a 63-year-old female with TI and HCV without cirrhosis, plus another 39-year-old male patient with TI and HCV associated with Child-Pugh-Class A cirrhosis. 

Data from the literature suggest a preventive effect of antiviral therapy on HCC development in patients with HCV-related cirrhosis [38,39], which is more evident in sustained virological responders [40]. However, HCC sometimes develops even after achieving viral eradication [41]. Interferon (IFN) therapy is a modality used to treat patient with chronic HCV infection, resulting in decreased intrahepatic inflammation, necrosis, and, consequently, decreased incidence of HCC [42,43]. Sustained HCV clearance obtained by interferon or pegylated interferon plus ribavirin therapy has been reported to significantly reduce HCC in HBV–HCV dually infected patients [44]. Even despite the high efficacy of the new direct-acting antiviral drugs in eliminating HCV hepatic infection, cases of HCC are still observed [45]. There are no data on the effect of anti-HCV treatment on HCC incidence in thalassemic patients. Ansari et al., in a single-center study, evaluated the incidence of HCC in 170 HCV RNA+ TM patients followed up for more than five years after IFN therapy [16]. 74% of the patients received IFN plus ribavirin, while the rest peg-IFN alone. All patients were on Deferoxamine therapy. HCC incidence was 0.6% (1/170). Conclusions definitely cannot be made about the effect of anti-HCV therapy on HCC incidence in thalassemia major patients from this report, but it would be interesting to have a larger study to assess that in the future. 

## 5. HCC in Thalassemia Risk Factors: Iron Overload

Iron overload is a well-known risk factor for multiple comorbidities including HCC [46]. The reported pooled incidence of HCC in hereditary hemochromatosis (HH) patients is around 5–10%, and this incidence can increase to 18% in the presence of cirrhosis [47,48]. In patients with ferroportin disease and African overload syndrome, after adjusting for alcohol consumption, hepatitis B, hepatitis C, and exposure to aflatoxin B1, patients with iron overload defined by transferrin saturation >60% and high ferritin, had a relative risk for HCC of 10.6 compared with those with normal iron stores [49]. Aside from its role in hepatic carcinogenesis through the induction of cirrhosis, there is substantial evidence to support the fact that iron overload may also increase the risk of HCC in the absence of cirrhosis, which plays a role in liver carcinogenesis in a complex and multifactorial manner [50,51,52]. In NTDT, iron overload is the result of hepcidin-mediated increased iron absorption from the gut, and increased recycling of iron from the reticuloendothelial system, leading to preferential iron loading of the hepatocytes [53]. This results in increased nontransferrin-bound iron in the circulation, leading to increased circulating free radicals and subsequent organ damage [54].

Several human, animal, and in vitro studies reported that excess iron can have a causative role in hepatic carcinogenesis [55]. This could be directly by effects on cellular proliferation, DNA interaction with the inactivation of tumor-suppressor genes, or indirectly through the formation of reactive oxygen species (ROS) and free radicals that can increase hepatic inflammation and fibrogenesis [46,56]. There is also evidence of possible immunologic role for iron overload leading to decreased immune surveillance for malignancy [57]. (Figure 1)

## 6. Intracellular Effects of Iron on Proliferation

There are experimental data revealing a role for iron in enhancing hepatic tumor growth, which was suppressed by deferoxamine [58,59]. This is possibly through the inhibition of the G1 phase [60]. Deferoxamine has been shown in some reports to have antitumor effects on leukemia and neuroblastoma cells [16] by likely affecting cell-cycle control and protecting against effects of oxygen free radicals [61]. It is also shown that dysplastic hepatic lesions in hemochromatosis patients, while they are iron-free lesions, use iron provided by the iron-overloaded normal milieu in their continued growth if they transform into HCC. However, inducing iron overload in normal animal models failed to promote hepatic carcinogenesis, thus implying that iron may have a promoting rather than an inducing role in liver carcinogenesis [58].

## 7. Intranuclear Effects of Iron

ROS and free radicals are byproducts of increased nontransferrin-bound free iron (NTBFI). These byproducts can cause direct chromosomal damage by inducing strand breaks and lead to chromosomal instability and subsequently mutations that could be tumor promoting [49,62,63]; one of which is p53 mutation which is frequently seen in HCC patients [64,65] but also in cancer-free patients with hemochromatosis [66]. Increased intracellular iron, via oxyradicals, plays a role in increased lipid peroxidation and impaired DNA repair. Nitric oxide, a byproduct of the enzyme nitric oxide synthase 2, is shown to be increased in noncancerous hepatic regeneration nodules in cancer-free patients in 8 out of 14 HH patients with HCC [67]. Nitric oxide, in animal models, was found to cause DNA damage and induce mutations by lipid peroxidation and nitrosamide deamination [68,69,70]. Moreover, nitric oxide was found to possibly play a role in the impairment of DNA repair via DNA glycosylase [71]. Studies on hemochromatosis patients have shown that lipid peroxidation may cause increased etheno-dG and -dA DNA adducts [72,73].

## 8. Effect of Iron on the Immune System

NTBFI and ferritin are associated with impaired immunity by impairing lymphocyte proliferation and tumoricidal activity of macrophages [58,74]. Iron has a critical role in the ribonucleotidase reductase activity necessary for lymphocyte activation [75]. Nontransferrin-bound iron may inhibit, in vitro, CD4 lymphocytes [57], a finding that may explain the presence of the low CD4 to CD8 ratio seen in thalassemic and genetic hemochromatosis patients in some series [76]. Moreover, lymphocyte proliferation has been found to be inhibited by ferritin [77,78] and mice macrophages exposed to iron salts, iron dextran, carbonyl iron, and iron-containing ferritin had suppressed tumoricidal activity.

Delayed initiation of iron chelation and poor compliance are major factors that leave thalassemic patients, especially TI who are nontransfusion-dependent at higher risk for developing HCC even in the absence of hepatitis infection or cirrhosis. In the initial Italian series in 2004 [17], out of the 22 HCC cases in thalassemia patients, two patients had no evidence of hepatitis but rather had grade-four siderosis in their liver. Table 1 summarizes the data on thalassemic patients with HCC who are hepatitis-negative. Of note is that the majority of these patients had poor compliance or erratic use of iron chelation. Moreover, despite the fact that many had lower serum ferritin at HCC diagnosis, they had significantly elevated serum ferritin years before their HCC diagnosis. This finding stresses the fact that it is likely chronic iron-overload exposure may predispose these patients to HCC. (Figure 1)

## 9. Management of HCC in Thalassemia Patients—Screening

The management of HCC in patients with thalassemia should be based on different approaches. It should start with an early screening for all patients, followed by prevention or control of major risk factors, especially for the high-risk groups (iron overload, hepatitis infection, cirrhosis), and ending up with proper medical or surgical treatment. Due to the prolonged survival seen nowadays in thalassemic patients, management should follow the same recommendation as in nonthalassemic patients with HCC.

Even though few data exist on the best modality for screening in thalassemia patients and lack of evidence of survival benefit due to the rarity of HCC in this group, establishing screening strategies for the early detection of risk factors or even HCC may prevent or effectively manage HCC. The importance of screening is reinforced by the fact that the majority of HCC cases are almost completely asymptomatic until advanced stages, as shown by the Italian registry of thalassemic patients in 2014, whereby around 82% of the 62 cases of HCC were asymptomatic or had nonspecific symptoms [15].

Therefore, we propose a two-step screening strategy. The first and key step includes screening all thalassemic patients for the presence of risk factors like iron overload, hepatitis viruses, and cirrhosis in an attempt to effectively manage these HCC risk factors in the future. The second step relies on screening those who are already in the high-risk group for the presence of HCC along with managing their risk factors.

Iron overload is the main risk factor seen in patients with thalassemia, thus a major contributor to many comorbidities including HCC. Often, iron overload is diagnosed late in the disease and, thus, screening for its presence is of utmost priority. 

First of all, iron overload should be clinically suspected, especially if recurrent transfusions exist. One of the early methods used to evaluate liver iron concentration (LIC) was liver biopsy [79]. This approach has almost been discarded, mainly due to its invasive nature and potentially serious complications related to the procedure. In addition, it has some limitations, especially in the difference seen in iron distribution in liver tissue [80].

Ferritin, on the other hand, being a less expensive and noninvasive option, is used as an indirect measure of iron overload by reflecting increased iron stores in the body [81,82]. However, it might not reflect the true burden of iron, specifically in NTDT patients where iron is accumulated in the hepatocytes [83]. In addition, ferritin is also a marker of inflammation and can increase in some inflammatory conditions and alcohol abuse [84].

For that, direct measure of LIC is a better reflection of iron burden and noninvasive quantification using R2 and R2* magnetic resonance imaging (MRI) is the mainstay test that should be used on a yearly basis to precisely monitor total body iron levels in all thalassemic patients [83].

Besides monitoring the iron load in patients with thalassemia, liver imaging and serum tumor markers also have a role in the early detection of HCC. The early detection of liver fibrosis, cirrhosis, or even carcinoma helps in improving patients’ overall survival [85]. Regular abdominal ultrasound for patients with cirrhosis, as frequent as twice yearly, is recommended by the American Association of the Study of Liver Diseases [10,86]. In contrast, alpha fetoprotein (AFP), which is the main tumor marker that arises in presence of HCC, is inadequate to be used in the screening process due to its low sensitivity and specificity. In the updated Italian registry in 2014, of the available AFP data of 45 thalassemic patients with HCC, 20 had normal AFP at diagnosis. In another series, normal AFP was found at diagnosis in 3 TI patients with HCC [15,18]. 

Finally, based on the current available evidence, it is proposed that thalassemia patients with concurrent HCV and/or HBV infection, having LIC ≥ 5 mg Fe/g dry weight (DW) in NTDT, LIC ≥ 7 mg Fe/g DW in TDT, serum ferritin ≥1000 ng/mL, or advanced cirrhosis are considered high-risk groups. Hereafter, screening with a biannual liver ultrasound should be done for the early detection of HCC [19,87].

## 10. Management of HCC in Thalassemia Patients—Prevention

Prevention of HCC essentially relies on the early management of the risk factors, namely, iron overload and hepatitis infections [3]. As for the former, intervention is based on using iron-chelation therapy aiming to decrease the iron burden and to prevent long-term complications as previously described. Three main chelating drugs are commercially available, deferoxamine, deferiprone, and deferasirox, with the latter being the only approved drug to be used for both TDT and NTDT [88]. The choice of treatment can be any of them, as a single agent or in combination. However, selection of best drug should be based on iron level, patient preferences, and compliance, taking into consideration their different toxicities [84]. The latest guidelines recommend treatment initiation in TDT whenever there is a ferritin level above 1000 ng/mL, LIC ≥ 7 mg Fe/g DW, and/or having received more than 10 blood transfusions. In contrast, lower cut-offs are proposed in NTDT due to the underestimation of the true iron burden in this category with recommendation being to start treatment when ferritin level exceeds 800 ng/mL or LIC ≥ 5 mg Fe/g dW [89,90].

In addition to managing iron overload, another major intervention relies on preventing infections with viral hepatitis [3] and treating them when indicated. This is accomplished by effective donor blood screening and by medically eradicating viral infections when indicated [91].

## 11. Management of HCC in Thalassemia Patients—Modalities of Treatment

Treatment options for HCC in thalassemia patient are largely based on data extrapolated from the general population due to the low incidence of HCC in thalassemia and to the limited data in this field. In fact, different approaches exist, and the best choice of treatment remains controversial, mainly due to the fact that the evidence behind it is only based on a series of case reports. The reported therapeutic modalities include surgical resection, chemoembolization, sorafinib, and percutaneous radioablation [3,21,22,92]. However, thalassemia patients with HCC should be managed similarly to their nonthalassemia counterparts.

On the other hand, liver transplantation is another proposed modality for the management of HCC in the general population [93]. For that, the selection of eligible patients is assessed using preset criteria, called Milan’s criteria, which include the size and number of the tumor lesions [94]. For patients with thalassemia, benefits from transplantation are not yet proven, given the fact that only a few patients underwent liver transplantation because it was contraindicated for thalassemic patients for a long while. Outcomes from the few published case reports showed promising results. Therefore, liver transplantation should be perceived as a possibly curative treatment in highly selected patients [3,15,24].

To note, dual organ transplantation may also be considered in any patient presenting with liver iron overload and severe cardiac failure in association with HCC to improve survival. Until now, only one case report has been published including a dual cardiac–liver transplantation in a thalassemic patient [95]. The crucial part is that transplantation should be done from the same donor, and finding the appropriate donor is not always easy.

## 12. Conclusions

Thalassemia patients are living longer and, despite the advent of effective hepatitis screening/treatment and iron-chelation therapies, many thalassemic patients are living longer with these comorbidities increasing the incidence of complications of their disease, including HCC. Many mechanisms have been described assessing the roles of hepatitis viruses, cirrhosis, transfusions, and, most importantly, iron overload in liver carcinogenesis. Effectively screening for these risk factors and timely managing them will most likely decrease the risk of HCC and improve survival. However, to date, there have been no randomized trials looking at these interventions in thalassemic patients. Screening with serial liver ultrasound biyearly for patients with risk factors, along with the less-sensitive AFP, continue to be the main modalities used nowadays until more sensitive modalities, like liver MRI, find their way into the guidelines.

## Figures and Tables

**Table 1:** Summary of data on hepatitis-negative thalassemic patients who developed hepatocellular carcinoma.

Patient	Sex	Age at Diagnosis	Phenotype	HCV Ab	HCV RNA	HBV Status	Serum Ferritin	Survival	Remarks
1	M	48	TI	−	−	−	1520 µg/L	alive at 16 months	Grade 4 hemosiderosis no cirrhosis [17]
2	F	61	TI	+	−	−	369 µg/L	Five months	Grade 4 hemosiderosis no cirrhosis [17]
3	M	22	TM	−	−	−	2000 µg/L	Five months	[17]
4	M	71	TI	−	−	−	600 µg/L	Five months	CPC B, Grade 6 fibrosis LIC 5.2 mg/g DW [18]
5	M	53	TI	−	−	−	1350 µg/L	Six months	CPC B, Grade 6 fibrosis LIC 4.8 mg/g DW [18]
6	F	41	TI	−	−	−	1450 µg/L	Three months	CPC B, Grade 5 fibrosis LIC 6.9 mg/g DW [18]
7	F	59	TI	−	−	NA	990 µg/L	25 months	no cirrhosis [24]
8	M	73	TI	−	−	NA	574 µg/L	Seven months	no cirrhosis [24]
9	M	54	TI	−	−	−	1291 µg/L	1 month	no cirrhosis, LIC 12.3 mg/g DW [19]
10	M	55	TI	−	−	−	5602 µg/L	48 months	no cirrhosis, LIC 23.9 mg/g DW Siderosis [19]
11	M	55	TI	−	−	−	832 µg/L	In remission	CPC B, LIC 1.19 mg/g DW 3 years ealrier: ferritin 3860 µg/L, LIC 7.3 mg/g DW [23]
4 patients	NA	NA	TI	−	−	−	1063–5678 µg/L	NA	[15]

M, male; F, female; TI, thalassemia intermedia; TM, thalassemia major; NA, not available; CPC, child-pugh-class; LIC, liver iron concentration; DW, dry weight; HCV Ab, hepatitis C virus antibody.
